# Association of mental health conditions and functional gastrointestinal disorders among Vietnamese new-entry medical students

**DOI:** 10.1371/journal.pone.0289123

**Published:** 2023-07-25

**Authors:** Tam Thao Tuyet Tran, Mai Ngoc Luu, Linh Le Tran, Duy Nguyen, Duc Trong Quach, Toru Hiyama

**Affiliations:** 1 Department of Family Medicine, University of Medicine and Pharmacy at Ho Chi Minh City, Ho Chi Minh City, Vietnam; 2 Department of Internal Medicine, University of Medicine and Pharmacy at Ho Chi Minh City, Ho Chi Minh City, Vietnam; 3 Department of General Surgery, Central Highlands Regional General Hospital, Buon Ma Thuot City, Daklak, Vietnam; 4 Health Service Center, Hiroshima University, Higashihiroshima, Japan; University of Malaya Faculty of Medicine, MALAYSIA

## Abstract

**Background:**

Functional gastrointestinal disorders (FGIDs), also known as disorders of gut-brain interaction, occur not only in the elderly but also in young adults. This study aimed to evaluate the association between mental health conditions and FGIDs among Vietnamese new-entry medical students.

**Methods:**

This cross-sectional study was conducted in February 2022 among new-entry medical students in Ho Chi Minh City, Vietnam. A printed questionnaire was distributed to all students on the day of freshmen health screening. Their urine samples were collected to screen for *Helicobacter pylori* infection using rapid urinary test. FGIDs were diagnosed using ROME IV criteria. Gastroesophageal reflux disease (GERD) was defined as the presence of typical reflux symptoms at least twice a week. Mental health conditions, including generalized anxiety disorder (GAD) and major depressive disorder (MDD), were identified using Generalized Anxiety Disorder Assessment-7 (GAD-7) and Patient Health Questionnaire-9 (PHQ-9) scales, respectively.

**Results:**

Among 400 new-entry medical students who participated in the study, the overall prevalence of FGIDs was 10.3% (functional dyspepsia 6.5%, irritable bowel disease 5.5%). The overlap syndrome (OS) of GERD-FGIDs or different FGIDs was present in 3.0% of participants. The prevalences of GAD and MDD were 6.8% and 10.2%, respectively. The urinary test was positive in 180 (45.0%) participants. In the multivariable logistic regression analysis, MDD was significantly associated with not only the risk of FGIDs (OR = 5.599, 95%CI: 2.173–14.430, p<0.001) but also the risk of OS (OR = 10.076, 95CI%: 2.243–45.266, p = 0.003).

**Conclusions:**

MDD is associated with FGIDs and OS among new-entry medical students.

## Introduction

Functional gastrointestinal disorders (FGIDs), also known as disorders of gut-brain interaction, are among the most prevalent chronic conditions, affecting up to 40% of the general population worldwide [1]. Common FGIDs include functional dyspepsia (FD) and irritable bowel syndrome (IBS) occur not only in the elderly but also in children, adolescents, and young adults. Although published studies from other Asian countries have shown a high prevalence of these FGIDs, their prevalence in the Vietnamese population is still unknown [2].

Understanding the characteristics of FGIDs in Vietnam is crucial to address the unique aspects of this condition in the local context. Vietnam is a low- and middle-income country with the highest age-standardized incidence rate of gastric cancer in Southeast Asia [3]. Additionally, the high prevalence of *Helicobacter pylori* (*H*. *pylori*) infection among Vietnamese population compared to other parts of Asia may play a role in the development and clinical presentation of FGIDs in Vietnam [3].

Several risk factors have been reported to be associated with the development of FGIDs, such as diet patterns, sleeping situations, and lifestyle habits [4, 5]. In addition, psychological disorders, including anxiety and depression, are the most prevalent psychiatric comorbidity in FGID patients [6]. However, due to cultural differences between countries, the association between mental health conditions and common FGIDs in the Vietnamese population has not been well understood, especially in the young adult population. Therefore, our study aimed to identify the association between mental health conditions and common FGIDs, including FD and IBS among Vietnamese new-entry medical students.

## Materials and methods

This cross-sectional study was designed in November 2021 and executed in February 2022 among Vietnamese first-year medical students at the University of Medicine and Pharmacy at Ho Chi Minh City, Vietnam. The study was undertaken in accordance with the latest version of the Helsinki declaration and the STROBE checklist in the S1 Checklist. A printed pre-designed questionnaire was distributed to all new-entry medical students on the day of freshmen health screening at the beginning of the academic year (February 25^th^, 2022). On the same day, we collected the participants’ urine samples to screen for *H*. *pylori* infection using rapid urinary test (Rapirun® H. Pylori Antibody Stick, Otsuka Pharmaceutical Co., Ltd, Tokyo, Japan). This urinary test has been validated in the Vietnamese population with the reported sensitivity and specificity of 84.7% and 89.9%, respectively [7]. Inclusion criteria were being aged ≥ 18 years old and completely filling out the survey questionnaire. We excluded those who had missing data or did not provide urine samples.

### Questionnaire design

The questionnaire used in this study was developed and distributed in Vietnamese, as all participants were native Vietnamese speakers. The questionnaire comprised the following five parts: (1) demographic characteristics including age, gender, height, weight, history of *H*. *pylori* infection and eradication (yes/no); (2) lifestyle habits covering breakfast habits, time for studying, extracurricular activities (yes/no), smoking (yes/no), alcohol intake (yes/no), living alone (yes/no); (3) sleeping habits including sleep timing, sleep duration, self-assessment of sleep quality (good/poor); (4) gastrointestinal symptoms including regurgitation, heartburn, epigastric pain, epigastric burning, postprandial fullness, early satiation, recurrent abdominal pain related to defecation or change in frequency or form of stool, the frequency and duration of these symptoms; and (5) mental health status evaluated by Generalised Anxiety Disorder Assessment (GAD-7) and Patient Health Questionnaire-9 (PHQ-9) scales, respectively. In this questionnaire, we used the Vietnamese versions of GAD-7 and PHQ-9, which have been validated in the Vietnamese population in previous studies [8, 9].

### Definitions

The participants were clinically diagnosed with common FGIDs, including FD and IBS, using Rome IV criteria [10]. The presence of *H*. *pylori* infection was not considered as an exclusion criterion for the diagnosis of FD, in line with the current guidelines [11, 12]. In addition, we also evaluated the presence of gastrointestinal reflux disease (GERD) among participants, taking into account the potential interactions and overlapping features between GERD and FGIDs. GERD was diagnosed based on the presence of typical reflux symptoms (regurgitation and/ or heartburn) at least twice a week, following the global guideline of the World Gastroenterology Organisation [13]. A score of 10 or greater on GAD-7 was considered the presence of generalized anxiety disorder (GAD), while a cut-off of 10 on PHQ-9 was used to diagnose major depressive disorder (MDD) [14, 15]. When used as a screening tool, the GAD-7≥10 cut-off point has a sensitivity of 89% and a specificity of 82% for diagnosing GAD [14]. The PHQ-9≥10 has a sensitivity of 85% and a specificity of 89% for the diagnosis of MDD [15].

We obtained written informed consent from all participants prior to data collection. Ethical approval was obtained from the Board of Ethics in Biomedical Research of the University of Medicine and Pharmacy at Ho Chi Minh City, Vietnam (ethical code numbered 227/DHYD-HDDD). The collected paper questionnaires were completely anonymous and not revealed to any external entity.

### Sample size calculation

The sample size for this study was calculated using the following formula:

$$N=\frac{Z^{2}\;P\;(1-P)}{d^{2}}$$

Where:

N represents the required sample size,

Z is the Z-score corresponding to the desired confidence level,

P is the estimated prevalence of the condition,

d is the desired margin of error.

Based on a similar study by Goyal *et al*., we selected a desired prevalence (P) of 0.27 [16]. Assuming a confidence level of 95% and a margin of error (d) of 0.05, a sample size of 303 participants was considered adequate.

### Statistical analysis

The collected data were organized in an Excel spreadsheet (Microsoft Corp., Redmond, Washington, USA), which was then analyzed using SPSS software version 20.0 (SPSS Inc., Chicago, IL). The descriptive statistics were performed using the Student’s T-test, Mann-Whitney U, Chi-square, and Phi and Crammer’s V tests. Multivariable logistic regression analysis was performed to explore the associated factors of FGIDs among medical students. A p-value of less than 0.05 was considered statistically significant.

## Results

### Characteristics of participants

A total of 419 Vietnamese new-entry medical students were enrolled for the academic year. All of them were invited to participate in the study. Among them, 400 (95.5%) students completed the survey questionnaire and provided urine samples for *H*. *pylori* screening. The median age of participants was 19 years (range 19–30). The male/female ratio was 1.6/1. 7% of participants reported having a past history of *H*. *pylori* infection and eradication. However, on the day of freshmen health screening, the urinary test to screen for *H*. *pylori* infection was positive in 180 samples (45.0%). Using GAD-7 and PHQ-9 scales, we identified up to 12.5% of new entry medical students affected with GAD and/or MDD. Table 1 summarizes the characteristics of participants, divided into FGIDs and non-FGIDs groups. These two groups were statistically different in the habit of sleeping after midnight and the presence of GAD and MDD (p<0.05).

**Table 1:** The characteristics of participants.

	Non-FGIDs (n = 359)	FGIDs (n = 41)	Total (n = 400)	p-value
Gender				0.411
• Male	225 (62.7)	23 (56.1)	248 (62.0)	
• Female	134 (37.3)	18 (43.9)	152 (38.0)	
Age (median, range)	19 (19–30)	19 (19–20)	19.00 (19–30)	0.269^b^
BMI (median, IQR)	21.64 (19.38–23.95)	21.09 (19.30–23.03)	21.55 (19.38–23.78)	0.126^b^
Past history of *H*. *pylori* infection and eradication	23 (6.4)	5 (12.2)	28 (7.0)	0.189^a^
Breakfast habit				0.440
• Everyday	204 (56.8)	19 (46.3)	223 (55.8)	
• Usually	105 (29.2)	15 (36.6)	120 (30.0)	
• Sometimes	50 (13.9)	7 (17.1)	57 (14.2)	
Study duration each day				0.718
• < 6 hours	61 (17.0)	9 (22.0)	70 (17.5)	
• 6–8 hours	173 (48.2)	18 (43.9)	191 (47.8)	
• > 8 hours	125 (34.8)	14 (34.1)	139 (34.8)	
Extracurricular activities	285 (79.4)	28 (68.3)	313 (78.2)	0.103
Smoking	3 (0.8)	0 (0.0)	3 (0.8)	1.000^a^
Alcohol taking	3 (0.8)	0 (0)	3 (0.8)	1.000^a^
Living alone	44 (12.3)	3 (7.3)	47 (11.8)	0.450^a^
Sleeping duration per day (hours) (median, IQR)	7 (6–8)	7 (6–8)	7.00 (6–8)	0.869^b^
Sleeping after midnight	191 (53.2)	31 (75.6)	222 (55.5)	**0.006**
Poor sleep quality	74 (20.6)	13 (31.7)	87 (21.8)	0.103
GAD	20 (5.6)	7 (17.1)	27 (6.8)	**0.013** ^a^
MDD	27 (7.5)	14 (34.1)	41 (10.2)	**<0.001** ^a^
Positive urinary test	162 (45.1)	18 (43.9)	180 (45.0)	0.881

BMI: body mass index; FGIDs: functional gastrointestinal disorders; IQR: interquartile range; GAD: generalized anxiety disorder; MDD: major depressive disorder.

^a^Fisher’s exact test

^b^Mann-Whitney U test.

### The prevalence of GERD and FGIDs among new-entry medical students

**Fig 1:**
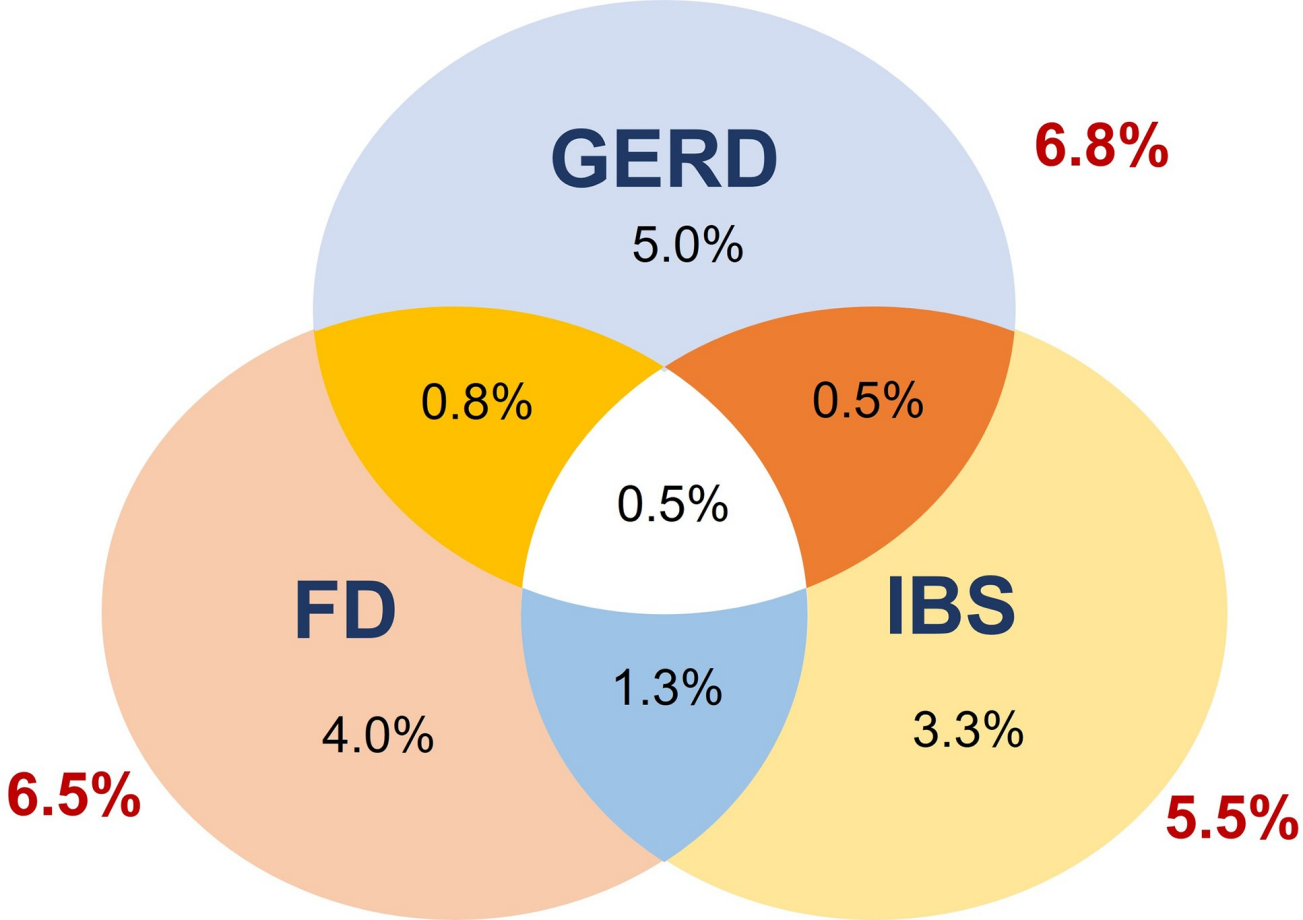
The prevalence of GERD and FGIDs among new-entry medical students. GERD: gastrointestinal reflux disease; FGIDs: functional gastrointestinal disorders, FD: functional dyspepsia; IBS: irritable bowel syndrome.

GERD and FGIDs (including FD and IBS) were common among new-entry medical students (Fig 1). The overall prevalence of FGIDs was 10.3%. The overlap syndrome (OS) of GERD-FGIDs or different FGIDs was present in 3.0% of participants. The prevalences of the overlap of GERD-FD, GERD-IBS, FD-IBS, and GERD-FD-IBS in the participants were 1.3%, 1.0%, 1.8%, and 0.5%, respectively.

### Association between mental health conditions and gastrointestinal disorders

Table 2 summarizes and compares the prevalences of gastrointestinal disorders according to each group of psychological diseases. The presence of gastrointestinal disorders was classified into three following groups: 1) meeting none of the criteria for GERD/FD/IBS; 2) meeting the criteria for only one gastrointestinal disorder (GERD, FD, or IBS); and 3) the overlap of GERD + FD, GERD + IBS, FD + IBS, and GERD + FD +IBS.

**Table 2:** Comparison of the gastrointestinal disorders according to each group of psychological diseases.

	Non-GAD (n = 373)	GAD (n = 27)	p-value*	Non-MDD (n = 359)	MDD (n = 41)	p-value^a^
Non-GERD/FD/IBS	318 (85.3)	21 (77.8)	0.276	306 (85.2)	33 (80.5)	0.423^b^
GERD alone	15 (4.0)	5 (18.5)	**0.007**	15 (4.2)	5 (12.2)	**0.043**
FD alone	14 (3.8)	2 (7.4)	0.295	11 (3.1)	5 (12.2)	**0.017**
IBS alone	10 (2.7)	3 (11.1)	**0.050**	10 (2.8)	3 (7.3)	0.138
Overlap GERD-FD	3 (0.8)	0 (0.0)	1.000	2 (0.6)	1 (2.4)	0.278
Overlap GERD-IBS	2 (0.5)	0 (0.0)	1.000	1 (0.3)	1 (2.4)	0.195
Overlap FD-IBS	4 (1.1)	1 (3.7)	0.296	3 (0.8)	2 (4.9)	0.084
Overlap GERD-FD-IBS	1 (0.3)	1 (3.7)	0.131	0 (0.0)	2 (4.9)	**0.010**

GERD: gastrointestinal reflux disease; FD: functional dyspepsia; IBS: irritable bowel syndrome; GAD: generalized anxiety disorder; MDD: major depressive disorder.

^a^Fisher’s exact test

^b^Chi-square test.

GERD alone was more common among individuals with GAD or MDD compared with those without these conditions (p< 0.01). The prevalence of FD alone was similar between GAD and non-GAD groups. However, FD alone was more common in students with MDD than non-MDD ones (12.2% vs. 3.1%, p = 0.017). On the other hand, IBS alone was present with equal frequency in the MDD and non-MDD groups but was more prevalent in the GAD group than in the non-GAD group (11.1% vs. 2.7%, p = 0.05). In addition, the overlap GERD-FD-IBS occurred more frequently among individuals with MDD (4.9% vs. 0%, p = 0.01).

### Associated factors of FGIDs among new-entry medical students in multivariable logistic regression model

After performing the multivariable logistic regression analysis, we found that the habit of sleeping after midnight and the presence of MDD significantly increased the odds of having FGIDs among new-entry medical students (p<0.05) (Table 3).

**Table 3:** Summary of multivariable logistic regression analysis for students with FGIDs.

Predictors	Multivariable		
	OR	95% CI	p-value
BMI	0.929	0.836–1.032	0.170
Past history of *H*. *pylori* infection and eradication	2.082	0.683–6.343	0.197
No extracurricular activities	0.540	0.252–1.158	0.114
Sleep after midnight	2.492	1.133–5.482	**0.023**
Poor sleep quality	1.322	0.579–3.018	0.501
GAD	1.232	0.388–3.914	0.723
MDD	5.599	2.173–14.430	**<0.001**

BMI: body mass index; OR: odd ratio; CI: confidence interval; GAD: generalized anxiety disorder; MDD: major depressive disorder.

### Associated factors of overlap gastrointestinal disorders among medical students in multivariable logistic regression model

The multivariable logistic regression analysis showed that female gender and the presence of MDD significantly increased the odds of having overlap gastrointestinal disorders among new-entry medical students (p<0.05) (Table 4).

**Table 4:** Summary of multivariable logistic regression analysis for students with overlap gastrointestinal disorders.

Predictors	Multivariable		
	OR	95% CI	p-value
Female gender	4.438	1.058–18.608	**0.042**
Past history of *H*. *pylori* infection and eradication	4.667	0.888–24.536	0.069
No extracurricular activities	0.325	0.085–1.237	0.099
Living alone	4.554	0.888–23.355	0.069
Sleep after midnight	7.530	0.845–67.132	0.071
Poor sleep quality	1.57	0.260–4.304	0.938
GAD	0.666	0.093–4.764	0.686
MDD	10.076	2.243–45.266	**0.003**

OR: odd ratio; CI: confidence interval; GAD: generalized anxiety disorder; MDD: major depressive disorder.

## Discussion

This study provided preliminary data on FGIDs based on Rome IV criteria in young Vietnamese adults considering the presence of mental health problems and other associated factors, including lifestyle habits, sleeping quality, and *H*. *pylori* infection status. The study participants were recruited as new-entry medical students and surveyed at the beginning of the school year to screen for gastrointestinal and psychiatric symptoms without being influenced by medical schooling.

In the current study, FGIDs were relatively common among Vietnamese new-entry medical students, with an overall prevalence for FGIDs of 10.3% (including 6.5% FD and 5.5% IBS). The prevalence of FGIDs in young populations based on Rome IV criteria varied greatly among different investigations. Studies conducted on young populations from Tunisia, Europe, and America reported the prevalences of FD and IBS of 5.0–6.7% and 3.0–7.6%, respectively [17, 18]. Few studies from Asia have been conducted on young populations examining the prevalence of FGIDs using the Rome IV criteria. A recent study conducted among Indian students reported a similar rate of IBS (6.2%) but a higher rate of FD (15.2%) compared to our findings [16]. This discrepancy may be due to the heterogeneity of the age of study populations and sociocultural differences between countries.

Among all FGIDs classified by the Rome Foundation, FD and IBS are the most broadly recognized and the most prevalent subtypes. These two conditions share common pathophysiological mechanisms, including altered gastric emptying, visceral hypersensitivity, post-infection dysbiosis, and increased mucosal permeability [19, 20]. The overlap FD-IBS and GERD-FGIDs have been well established in population-based studies and appear to be distinct entities, although they have not been specifically defined in the Rome I-IV criteria [21–24]. Our study showed that the OS of FD-IBS and GERD-FGIDs were present in 3.0% of participants, including the overlap of GERD-FD (1.3%), GERD-IBS (1.0%), FD-IBS (1.8%), and GERD-FD-IBS (0.5%). This result accords with the comorbidity of GERD and FGIDs in a Chinese rural population with a prevalence of 1.2% [22]. In a retrospective cohort study of 29,553 outpatients in Germany, 6% and 3% of IBS patients were diagnosed with FD 12 months before and after the time-point of IBS diagnosis, respectively [25]. Jones *et al*. found that 2% of an Australian random community sample met the criteria for overlap GERD-FD-IBS, which was estimated to be twenty-fold greater than the expected probability by random chance [24]. Although the prevalences of OS in Asian countries are generally lower than in Western countries, these findings confirm that OS in the general population is common and does not occur by chance.

A meta-analysis published in 2015 indicated that mental disorders were frequent among children and adolescents across 27 countries of all continents, with the worldwide pooled prevalence of any anxiety disorders and MDD of 6.5% and 1.3%, respectively [26]. Adolescents transitioning toward young adulthood were more vulnerable to mental illness than younger adolescents [27]. In the present study, the prevalences of GAD and MDD among new-entry students were 6.8% and 10.2%, respectively. Similarly, reports from the WHO World Mental Health International College Student project, which surveyed 19 colleges in Australia and Western countries, demonstrated that MDD was the most common mental disorder among first-year college students (12-month prevalence of 18.5%), followed by GAD (16.7%) [28]. However, cross-national studies using the same instrument and sampling technique showed a lower prevalence of anxiety and depression in Asian populations in comparison with Western populations [29, 30]. In this study, the prevalence of MDD among freshmen students was notably higher than that of the general Vietnamese population (2.45%) and those reported in other studies from Asia (4%) [31, 32]. This might be because the present study was conducted after the third wave of COVID-19 pandemic in Vietnam, which might increase the prevalence of MDD among the young population. Also, the recruited participants of our study were new-entry medical students; as a result, we should be cautious when extrapolating the conclusions to Vietnamese young adults. However, this high MDD rate is an alarming issue for the mental health of future medical staff in Vietnam. Further research is needed to screen and manage mental health problems among Vietnamese medical students.

The relationship between gastrointestinal disorders and mental health conditions has been well-recognized in previous studies. Systematic reviews demonstrated that the prevalence of anxiety and depression was higher in patients with IBS as compared with healthy subjects, regardless of the IBS subtype [33–35]. Similarly, FD patients were reported to have higher rates of anxiety and depression [36, 37]. However, these studies did not differentiate between patients with only one FGID or with overlapped gastrointestinal disorders, which may bias the real effects of mental health disorders on the presence of FGIDs. Our findings showed that MDD was significantly related to GERD alone, FD alone and overlap GERD-FD-IBS but not to IBS alone. On the contrary, GAD was significantly associated with GERD alone, IBS alone but not FD alone nor OS. Our study also revealed that MDD but not GAD was independently associated with FGIDs and OS. In an early study in Korea, Lee *et al*. reported that depressive mood was significantly associated with the presence of FD and overlap FD-IBS, but not that of IBS using Rome III criteria [38]. A more recent study in Korea using Rome IV criteria also observed that FD-IBS overlap patients had a higher prevalence of depression compared to non-overlap ones [39]. The different associations of anxiety and depression on these digestive disorders may be related to diverse biological mechanisms and different interactions of neurotransmitters on intestinal motility. Functional neuroimaging studies revealed that patients with FD or IBS had activity changes in several brain areas, including the prefrontal cortex, anterior cingulate cortex, somatosensory cortex, insula, amygdala, hippocampus, and thalamus [40–42]. These brain areas are involved in the sensory signal reception from the digestive tract and the information procession of visceral sensation. Moreover, the prefrontal cortex regulates emotions associated with pain, the anterior cingulate cortex acts as a mechanism to screen or process unpleasant sensations, and the amygdala assumes a critical role in modulating sensations related to stress and pain [40]. However, very few studies have evaluated the alterations of brain structures associated with OS. Further prospective research is needed to clarify our understanding of the role of mental health and FGIDs, particularly for the overlap gastrointestinal disorders.

In the present study, the female gender significantly increased the odds of having OS. Previous studies have found an association between FGIDs and mental health for women [43]. The increased risk of all gastrointestinal disorders, including GERD, IBS and dyspepsia, was reported to be greatest among women with depression [43]. Women with FD-IBS overlap were reported to present more severe gastrointestinal and depression symptoms than men [39]. A systematic review of the biological sex differences in MDD found that depressed females had significantly higher levels of inflammatory, serotonergic, and neurotrophic markers compared with depressed males [44]. In addition, the severity of depressive symptoms in women correlated with the levels of some inflammatory and neurotrophic factors [44]. There are several hypothesized mechanisms for interpreting sex differences in the OS, such as the differences in sex hormones, stress response, gastrointestinal motility, gut microbiota, visceral perception, and inflammatory cytokine expression between males and females [39, 45, 46]. Further studies are needed to confirm the association between gender and OS.

Furthermore, our study revealed a significant association between FGIDs and the habit of sleeping after midnight. This association is consistent with previous research showing that disrupted circadian rhythms, irregular sleep patterns, inadequate sleep, late-night eating, exposure to stressors during late hours, and the influence of psychological factors on sleep disturbances may contribute to the development and exacerbation of FGID symptoms [47–51]. These findings support the growing recognition of the role of sleep in gastrointestinal function and symptomatology [51].

In addition to exploring the association between FGIDs and mental health conditions, it is important to address the high positivity rate (45%) on the urine test for *H*. *pylori* observed in our study. This finding aligns with previous community-based studies in Vietnam, which reported a *H*. *pylori* prevalence ranging from 34% to 89% among children and 58% to 75% in the general population [52–55]. This indicates a significant burden of *H*. *pylori* infection in Vietnam. Further research is needed to explore its implications for FGIDs and mental health in Vietnamese young adults.

The present study had several limitations. First, the diagnosis of FGIDs and GERD was primarily based on clinical criteria. Structural diseases or other underlying pathologies that can mimic FGIDs or GERD were not excluded through endoscopy or pH monitoring. Second, this study collected answers from participants using a questionnaire; as a result, the responses might be influenced by recall bias. Additionally, the categorization of participants into GAD and MDD groups, based on validated screening tools, has the potential for overestimation of symptoms given the significant life changes and adjustments experienced by students during the transition to university. This highlights the need for careful consideration and interpretation of our findings, taking into account the context of the college transition and the potential impact it may have on mental health assessments. Third, although the study was conducted at the beginning of the school year, all participants were medical students, which may not reflect the real picture of the FGID burden among the Vietnamese young adult population. Furthermore, since the study was conducted after the COVID-19 pandemic wave in Vietnam, the study’s results regarding the prevalence of FGIDs and mental health conditions might have been affected. Lastly, the sample size of participants with OS, a subgroup of interest, was relatively small, which may have limited the power to detect significant associations with conditions such as MDD. Future studies with a larger sample size are needed to enable a more comprehensive analysis of the association between MDD and OS.

## Conclusions

The present study is the first observational study in Vietnam to examine the prevalence and associated factors of FGIDs using the Rome IV criteria in the young adult population. Our findings suggest that MDD is associated with FGIDs and OS among new-entry medical students.

## Supporting information

S1 Checklist STROBE checklist. (DOCX)

S1 Data Data set. (XLSX)
